# Cisplatin Induces Inflammation by Activating IL-6 via Suppressing rno-let-7g-5p and rno-let-7f-5p Expression in Intestinal Epithelial Cells

**DOI:** 10.7150/ijms.131043

**Published:** 2026-03-17

**Authors:** Chi-Jen Chang, Tsung-Ming Chang, Ying-Sui Sun, Kuan-Ting Lu, Ju-Fang Liu

**Affiliations:** 1 School of Medicine, Fu Jen Catholic University, No. 510, Zhongzheng Rd., Xinzhuang Dist., New Taipei City 242062, Taiwan.; 2 Division of Pediatric Surgery, Shin Kong Wu Ho-Su Memorial Hospital, No. 95, Wenchang Rd., Shilin Dist., Taipei City 111045, Taiwan.; 3 School of Dental Technology, College of Oral Medicine, Taipei Medical University, No. 250, Wuxing St., Xinyi Dist., Taipei City 110301, Taiwan.; 4 Department of Neurosurgery, Shin Kong Wu Ho-Su Memorial Hospital, No. 95, Wenchang Rd., Shilin Dist., Taipei City 111045, Taiwan.; 5 Translational Medicine Center, Shin Kong Wu Ho-Su Memorial Hospital, No. 95, Wenchang Rd., Shilin Dist., Taipei City 111045, Taiwan.; 6 School of Oral Hygiene, College of Oral Medicine, Taipei Medical University, No. 250, Wuxing St., Xinyi Dist., Taipei City 110301, Taiwan.; 7 Department of Medical Research, China Medical University Hospital, China Medical University, No. 91, Xueshi Rd., North Dist., Taichung City 404328, Taiwan.

**Keywords:** cisplatin, intestinal epithelial cells, interleukin-6, rno-let-7f-5p, rno-let-7g-5p, microRNA

## Abstract

Cisplatin is a widely used chemotherapeutic agent; however, its therapeutic efficacy is often limited by severe cytotoxic side effects, particularly gastrointestinal toxicity, which manifests as intestinal mucositis. MicroRNAs (miRNAs) are small non-coding RNAs that play critical roles in both normal physiological and pathological processes by regulating gene expression. However, their role in cisplatin-induced gastrointestinal toxicity remains unclear. In this study, we investigated the regulatory effects of miRNAs on cisplatin-induced inflammation in intestinal epithelial cells (IEC-6). Our results demonstrate that cisplatin significantly decreases cell viability while inducing interleukin-6 (IL-6) expression in a dose-dependent manner. Moreover, we observed that cisplatin activates the phosphorylation of p38 and ERK but does not activate JNK in IEC-6 cells. Using specific inhibitors of p38 and ERK, we confirmed their roles in regulating IL-6 expression. Through analysis of the miRNA database, we identified several miRNAs that potentially target IL-6. Notably, rno-let-7f-5p and rno-let-7g-5p showed significant downregulation following cisplatin treatment. Transfection mimics and inhibitors of rno-let-7f-5p and rno-let-7g-5p further confirmed their regulatory role in IL-6 expression. Importantly, inhibition of the p38 and ERK pathways attenuated the cisplatin-induced reduction of rno-let-7f-5p and rno-let-7g-5p levels, suggesting a potential regulatory link between MAPK signaling and miRNA expression. In conclusion, our findings support a model in which cisplatin promotes inflammation in intestinal epithelial cells by activating the p38 and ERK pathways and is associated with the suppression of rno-let-7f-5p and rno-let-7g-5p. These findings provide mechanistic rationale that may inform future efforts to mitigate cisplatin-associated gastrointestinal toxicity.

## Introduction

Cisplatin is a platinum-based chemotherapeutic agent extensively used to treat various cancers. Its efficacy is attributed to its ability to induce DNA damage, leading to apoptosis in cancer cells 1, 2. However, its clinical application is frequently constrained by cytotoxic side effects, including nephrotoxicity, ototoxicity, and gastrointestinal toxicity 1, 3-7. Among these, cisplatin-induced oxidative stress and inflammation in intestinal epithelial cells are particularly concerning, as they can compromise the integrity of the gut barrier and lead to intestinal mucositis 8-11. These side effects often necessitate interruption of treatment or dosage adjustments, thereby reducing the overall effectiveness of chemotherapy.

Interleukin-6 (IL-6) plays a crucial role in developing chemotherapy-induced intestinal mucositis, where it orchestrates inflammation and contributes to mucosal injury 12. IL-6 disrupts the intestinal epithelial barrier, increasing permeability and exacerbating tissue damage 10. Additionally, IL-6 modulates immune cell dynamics, which are essential to the tissue repair process. Emerging studies suggest that modulating IL-6 concentrations may reduce the severity of mucositis by diminishing inflammatory responses and preserving mucosal integrity 9, 10.

MicroRNAs (miRNAs) are small non-coding RNAs that play a crucial role in the post-transcriptional regulation of gene expression. They accomplish this by annealing to complementary sequences on target mRNAs, which suppresses protein production and subsequent modulation of cellular activities 13, 14. This regulatory function is vital in both normal physiological and pathological processes. Aberrant miRNA expression can disrupt cellular proliferation, differentiation, and even the effectiveness of therapeutic interventions 15, 16. In the context of intestinal epithelial cells, miRNAs are crucial for responding to external stressors and maintaining homeostasis following injury 14, 17. However, the mechanism by which cisplatin induces IL-6 expression through miRNA regulation remains unclear. This highlights the necessity of investigating effective auxiliary methods to mitigate the adverse effects on intestinal epithelial cells after cisplatin administration by modulating miRNA expression.

Despite evidence that cisplatin can trigger intestinal inflammation and that MAPK signaling and let-7 family miRNAs are each implicated in inflammatory regulation, how these pathways converge in intestinal epithelial cells to control IL-6 induction remains incompletely understood. Therefore, this study aimed to investigate the relationship among cisplatin exposure, MAPK activation, and rno-let-7f-5p/rno-let-7g-5p regulation in intestinal epithelial cells, and to determine whether changes in these miRNAs are associated with IL-6 upregulation. Using IEC-6 cells, we examined cisplatin-induced IL-6 expression, MAPK pathway activation, and the effects of pharmacological inhibition of p38/ERK/JNK as well as rno-let-7 gain- and loss-of-function on IL-6 responses.

## Materials and Methods

### Chemicals and reagents

Anti-rabbit IgG-conjugated horseradish peroxidase, along with rabbit polyclonal antibodies specific for IL-6, β-actin, p38, phospho-p38, ERK, phospho-ERK, JNK, and phospho-JNK, as well as goat anti-rabbit IgG, were obtained from GeneTex (GeneTex, USA). All other reagents were obtained from Sigma-Aldrich (Merck, USA).

### Cell cultures

Normal IEC-6 rat small intestine epithelial cells (BCRC Number: 60301) were obtained from Taiwan's Biological Resources Preservation and Research Center (BCRC). The cells were cultured in DMEM supplemented with 10% fetal bovine serum (FBS, Thermo Fisher, USA), 2 mM GlutaMAX-I (Thermo Fisher, USA), 100 units/ml penicillin, and 100 μg/ml streptomycin (Thermo Fisher, USA), with the pH adjusted to 7. Cultures were maintained at 37°C in a 5% CO_2_ atmosphere, replenishing the medium every two days.

### Western blot analysis

Proteins were separated using 10-15% SDS-PAGE and subsequently transferred to 0.45-μm PVDF membranes (Merck, USA). Membranes were blocked with 5% non-fat milk at room temperature for 1 hour, followed by incubation with primary antibodies at a 1:1,000 dilution overnight at 4°C. After three washes with TBST, membranes were incubated with horseradish peroxidase-conjugated secondary antibodies at a 1:10,000 dilution for 1 hour at room temperature. Protein bands were detected using enhanced chemiluminescence (RPN2235, Amersham™ Cytiva, USA) and visualized with a chemiluminescence imaging system (UVP ChemiDOC-It 815, Analytik Jena, USA).

### Quantitative real-time polymerase chain reaction (qPCR)

Total RNA was extracted from IEC-6 cells using the easy-BLUE Total RNA Extraction Kit (iNtRON Biotechnology, Korea). First-strand cDNA synthesis for mRNA analysis was conducted using the qPCRBIO cDNA Synthesis Kit (Cat. #PB30.11-10, PCR Biosystems, UK), and miRNA cDNA synthesis was performed using the Mir-X miRNA First-Strand Synthesis Kit (Cat. #638313, Takara, Japan). The expression of rat IL-6, β-actin, rno-let-7a-5p, rno-let-7b-5p, rno-let-7c-5p, rno-let-7e-5p, rno-let-7i-5p, rno-miR-98-5p, rno-miR-149-5p, and U6 was quantified using the KAPA SYBR FAST qPCR Master Mix (2×) (Sigma-Aldrich) and the CFX Connect Real-Time PCR Detection System (BioRad, Hercules, USA), following the manufacturer's protocol. Primers were obtained from MDBio, Inc. (MDBio, Inc., New Taipei City, TW). Gene expression levels were quantified using the 2^-ΔΔCt^ method and normalized against β-actin and U6 as internal controls.

### Transfection of microRNA mimics and inhibitors

IEC-6 cells (2×10^5^) were transfected with 50 nM of rno-rno-let-7f-5p or rno-rno-let-7g-5p mimics, hairpin inhibitors, and corresponding negative controls (miRIDIAN, Horizon, US) for 24-hour. The sequences used were: rno-rno-let-7f-5p mimic (5'-UGAGGUAGUAGAUUGUAUAGUU-3'), rno-rno-let-7f-5p inhibitor (5'-AACUAUACAAUCUACUACCUCU-3'), rno-rno-let-7g-5p mimic (5'-UGAGGUAGUAGUUUGUACAGUU-3'), rno-rno-let-7g-5p inhibitor (5'-AACUGUACAAACUACUACCUCU-3'), mimic negative control (5'-UUGUACUACACAAAAGUACUG-3'), and inhibitor negative control (5'-UCACAACCUCCUAGAAAGAGUAGA-3'). Post-transfection, cells were treated with cisplatin, and IL-6 expression was assessed via qPCR. Each experiment was performed in triplicate in three independent experiments.

### Reporter gene assay

IEC-6 cells were seeded at a density of 1×10^5^ cells per well in 12-well plates. Each well (containing 1×10⁵ IEC-6 cells) was transfected with 1 μg of an IL-6 3'UTR wild-type plasmid or mutant IL-6 3'UTR plasmid (MDBio, Taiwan), with or without 50 nM rno-rno-let-7g-5p mimic, using Lipofectamine 3000 (Thermo Fisher, USA). The DNA-to-reagent ratio was maintained at 1:2 (µg: µL). Following transfection, cells were treated with cisplatin for 24-hour. Cells were lysed using 100 μL of reporter lysis buffer (E153A, Promega, Madison, WI, USA), and lysates were centrifuged at 13,200 rpm for 15 minutes. 50 μL aliquot of each lysate was transferred to an opaque white 96-well microplate, and 50 μL of luciferase assay substrate (Luciferase Assay System E151A, Promega, Madison, WI, USA) was added. Relative luciferase activity was measured using a VICTOR X2 microplate luminometer (PerkinElmer, MA, USA).

### Statistical analysis

Data are presented as the mean ± standard deviation (SD). For multi-group comparisons, one-way ANOVA followed by Tukey's multiple comparisons test or two-way ANOVA followed by Sidak's multiple comparisons test was applied, as appropriate. Statistical significance was defined as a *p*-value <0.05.

## Results

### Cisplatin induces IL-6 expression and compromises cell viability in IEC-6 cells

**Figure 1:**
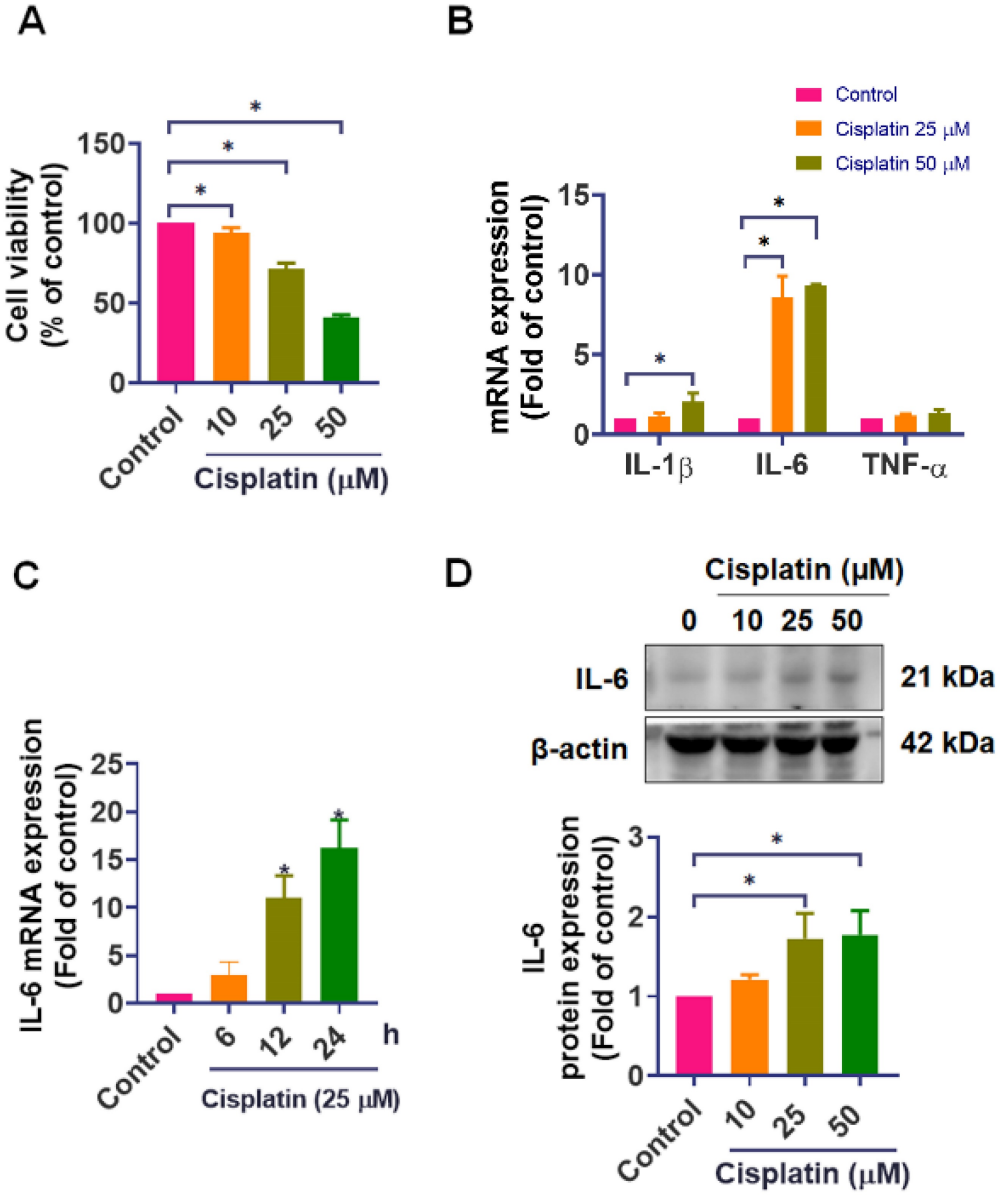
** Effect of cisplatin on cell viability and IL-6 expression in IEC-6 cells. (A)** IEC-6 cells were treated with cisplatin (0, 10, 25, 50 μM) for 24-hour (n = 4), and cell viability was measured using the CCK-8 assay. **(B)** IL-1β, IL-6, and TNF-α mRNA levels were quantified by real-time PCR following 24-hour cisplatin treatment (n=4). **(C)** Time-course analysis of IL-6 mRNA expression at 0, 6, 12, and 24-hour after cisplatin exposure (n = 4).** (D)** IL-6 protein expression was evaluated by Western blot analysis (n = 4). Cells without treatment served as the control group. Data are presented as means ± SD. Significance is denoted by *p < 0.05.

In our investigation, we established the detrimental effects of cisplatin on IEC-6 cells. We treated IEC-6 cells with different concentrations of cisplatin (0, 10, 25, and 50 μM) for 24-hour, cell viability was subsequently assessed using the CCK-8 assay. The results revealed a significant decline in the survival rate of IEC-6 cells (25 μM: 71.5%, and 50 μM: 40.7%) in response to cisplatin treatment (Figure 1A). We next examined the expression of key inflammatory cytokines. qPCR analysis revealed that cisplatin markedly upregulated IL-6 mRNA, showing an 8.6-fold and 9.4-fold increase at 25 μM and 50 μM, respectively, compared with untreated cells. Cisplatin at 50 μM also increased IL-1β mRNA by approximately 2-fold, while tumor necrosis factor-α (TNF-α) mRNA levels remained unchanged (Figure 1B). To better characterize the temporal dynamics of the inflammatory response, we performed a time-course analysis of IL-6 expression at 0, 6, 12, and 24-hour after cisplatin treatment. IL-6 mRNA gradually increased and reached its maximum level at 24-hour (Figure 1C). Additionally, cisplatin treatment elevates IL-6 protein levels (Figures 1D). These findings confirm that cisplatin upregulates IL-6 expression, which is closely associated with its cytotoxic impact on intestinal epithelial cells, triggering an inflammatory cellular response.

### Cisplatin-induced IL-6 expression by p38 and ERK activation

**Figure 2:**
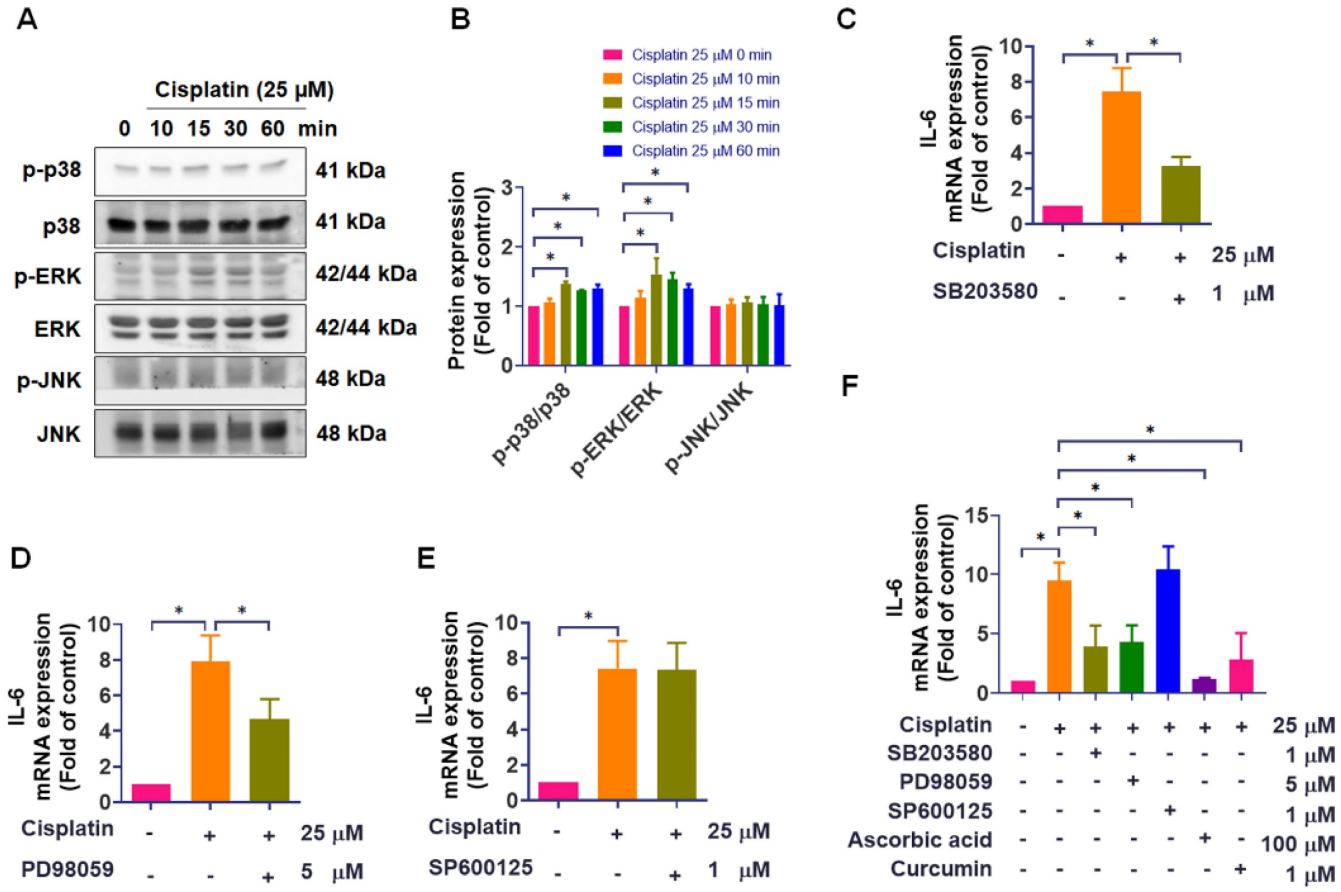
** The role of MAPK signaling in cisplatin-induced IL-6 expression. (A)-(B)** IEC-6 cells were treated with 25 μM cisplatin for 0-60 min, and phosphorylation of p38, ERK, and JNK was analyzed by Western blot (n = 4). **(C)-(E)** IEC-6 cells were pretreated with SB203580 (p38 inhibitor, 1 μM), PD98059 (ERK pathway inhibitor, 5 μM), or SP600125 (JNK inhibitor, 1 μM) for 1-hour, followed by cisplatin (25 μM) treatment for 24-hour. IL-6 mRNA expression was quantified by real-time PCR (n = 4). **(F)** Using the same protocol, cells were pretreated with SB203580, PD98059, SP600125, ascorbic acid (IL-6 inhibitor, 100 μM), or curcumin (IL-6 inhibitor, 1 μM) prior to cisplatin treatment, and IL-6 mRNA was quantified by real-time PCR (n = 4). Cells without treatment acted as the control group. Data are expressed as means ± SD. Significance is denoted by *p < 0.05.

The mitogen-activated protein kinase (MAPK) signaling pathway is a critical regulator of inflammatory responses, such as intestinal mucositis 18-22. To explore whether MAPK signaling may participate in the upstream regulation of IL-6, we performed pathway analysis using the MetaCore online platform. Our study observed that treatment with cisplatin for 15 minutes significantly enhanced the phosphorylation of p38 and ERK, continuing until 60 minutes, while JNK phosphorylation was unaffected (Figure 2A-B). We next assessed the functional involvement of each MAPK branch using specific inhibitors. Pretreatment with the p38 inhibitor SB203580 (1 μM) or the ERK pathway inhibitor PD98059 (5 μM) significantly attenuated cisplatin-induced IL-6 mRNA expression. However, the JNK inhibitor SP600125 (1 μM) failed to reduce IL-6 levels (Figure 2C-E). To further validate these findings, we used two well-established IL-6-suppressing agents, ascorbic acid and curcumin, as positive controls 23-27. Both compounds markedly inhibited cisplatin-induced IL-6 expression, confirming the responsiveness of our detection system. Consistent with our inhibitor data, SB203580 and PD98059 reduced IL-6 expression, whereas SP600125 showed no inhibitory effect even under conditions where positive controls were effective (Figure 2F). The inhibitory effects of SB203580 and PD98059 were further confirmed by Western blot assay (Figure S1). These findings indicate that, under our experimental conditions, cisplatin-induced IL-6 upregulation in IEC-6 cells is associated with activation of the p38 and ERK pathways, whereas JNK activation was not detectably increased and JNK inhibition did not attenuate IL-6 induction. This conclusion is further supported by supplemental data showing that SB203580 and PD98059 effectively suppressed cisplatin-induced p38 and ERK phosphorylation (Figure S1).

### Cisplatin downregulates rno-let-7f-5p and rno-let-7g-5p targeting IL-6 in IEC-6 cells

**Figure 3:**
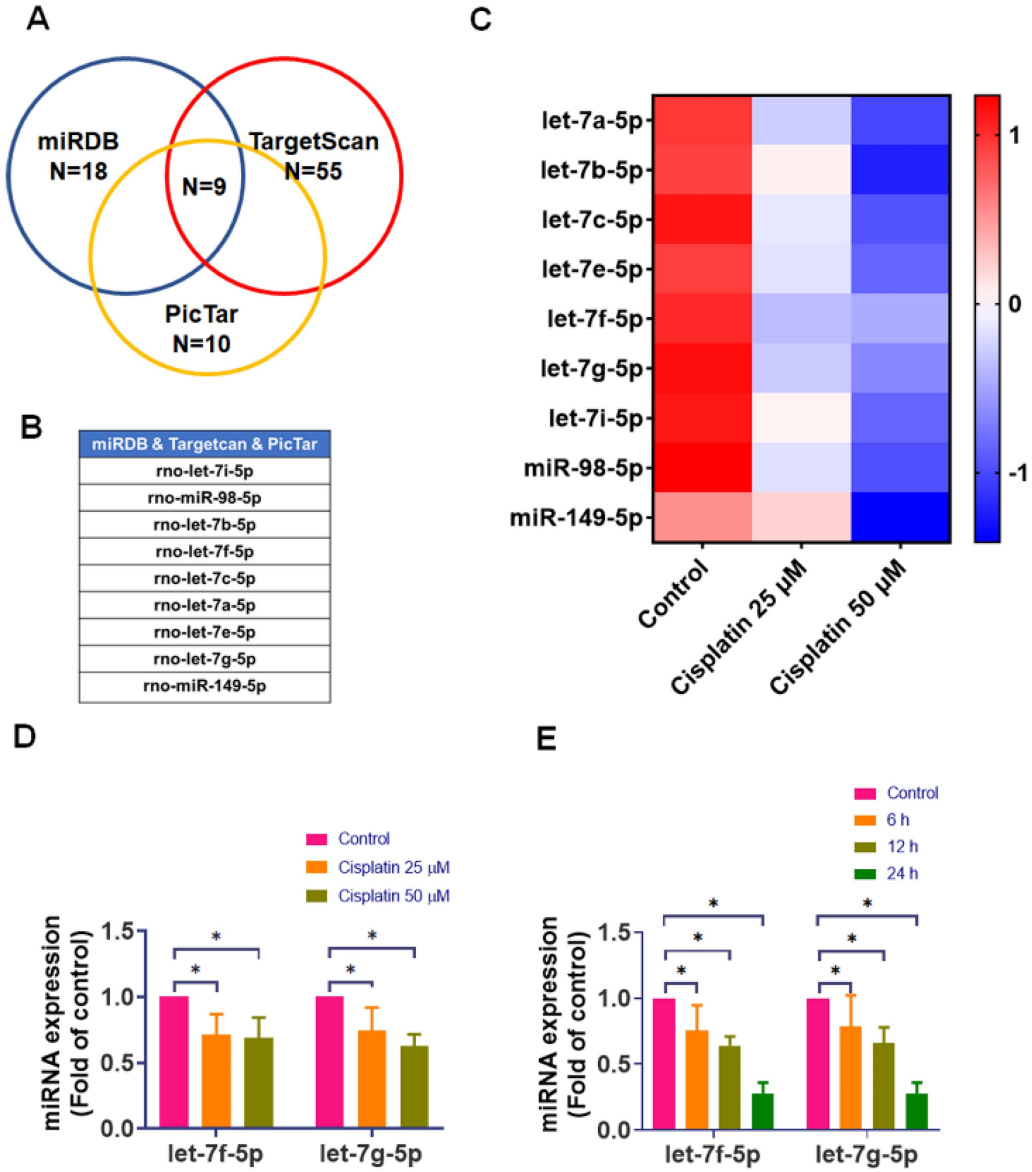
** Effect of cisplatin on IL-6-targeting miRNA expression in IEC-6 cells. (A)** Identification of potential IL-6-targeting miRNAs using Venn diagrams to compare predictions across different databases. **(B)** List of candidate miRNAs predicted to target IL-6. **(C)** IEC-6 cells were exposed to cisplatin (0-50 μM) for 24-hour (n = 4). Expression levels of candidate miRNAs were quantified by real-time PCR, normalized as Z-scores, and displayed as a heat map.** (D)** Expression of rno-let-7f-5p and rno-let-7g-5p was measured by real-time PCR after 24-hour of cisplatin treatment (0-50 μM) (n = 4). **(E)** Time-course analysis of rno-let-7f-5p and rno-let-7g-5p expression after cisplatin exposure for 0-24-hour (n = 4). Cells without treatment acted as the control group. Data are expressed as means ± SD. Significance is denoted by *p < 0.05.

miRNAs are recognized as critical regulators of cellular protein expression 28. To identify candidate miRNAs that may regulate IL-6 in IEC-6 cells, we first performed *in silico* target prediction using three public databases (miRDB, TargetScan, and PicTar). This search yielded 18 miRNAs in miRDB, 55 in TargetScan, and 10 in PicTar that were predicted to target IL-6. To prioritize high-confidence candidates and reduce false-positive predictions, we focused on miRNAs that were consistently predicted across all three platforms; the intersection analysis identified nine overlapping candidates (Figure 3A-B). We then conducted an experimental screening step by quantifying the expression of these nine candidates in IEC-6 cells after cisplatin treatment (0, 25, and 50 μM for 24-hour) using qPCR. Heat map visualization revealed a general downregulation pattern among the candidate miRNAs following cisplatin exposure (Figure 3C). Notably, rno-let-7f-5p and rno-let-7g-5p showed a prominent and reproducible reduction with clear dose dependence, displaying clear dose-dependent suppression (Figure 3D). Because these two miRNAs exhibited the strongest cisplatin responsiveness among the high-confidence candidates, we selected them for further characterization. Consistently, time-course analysis demonstrated that cisplatin progressively reduced rno-let-7f-5p and rno-let-7g-5p levels, with maximal suppression at 24-hour (Figure 3E). Collectively, these results indicate that cisplatin downregulates the IL-6-targeting miRNAs rno-let-7f-5p and rno-let-7g-5p in IEC-6 cells, supporting their potential involvement in cisplatin-associated IL-6 upregulation.

**Figure 4:**
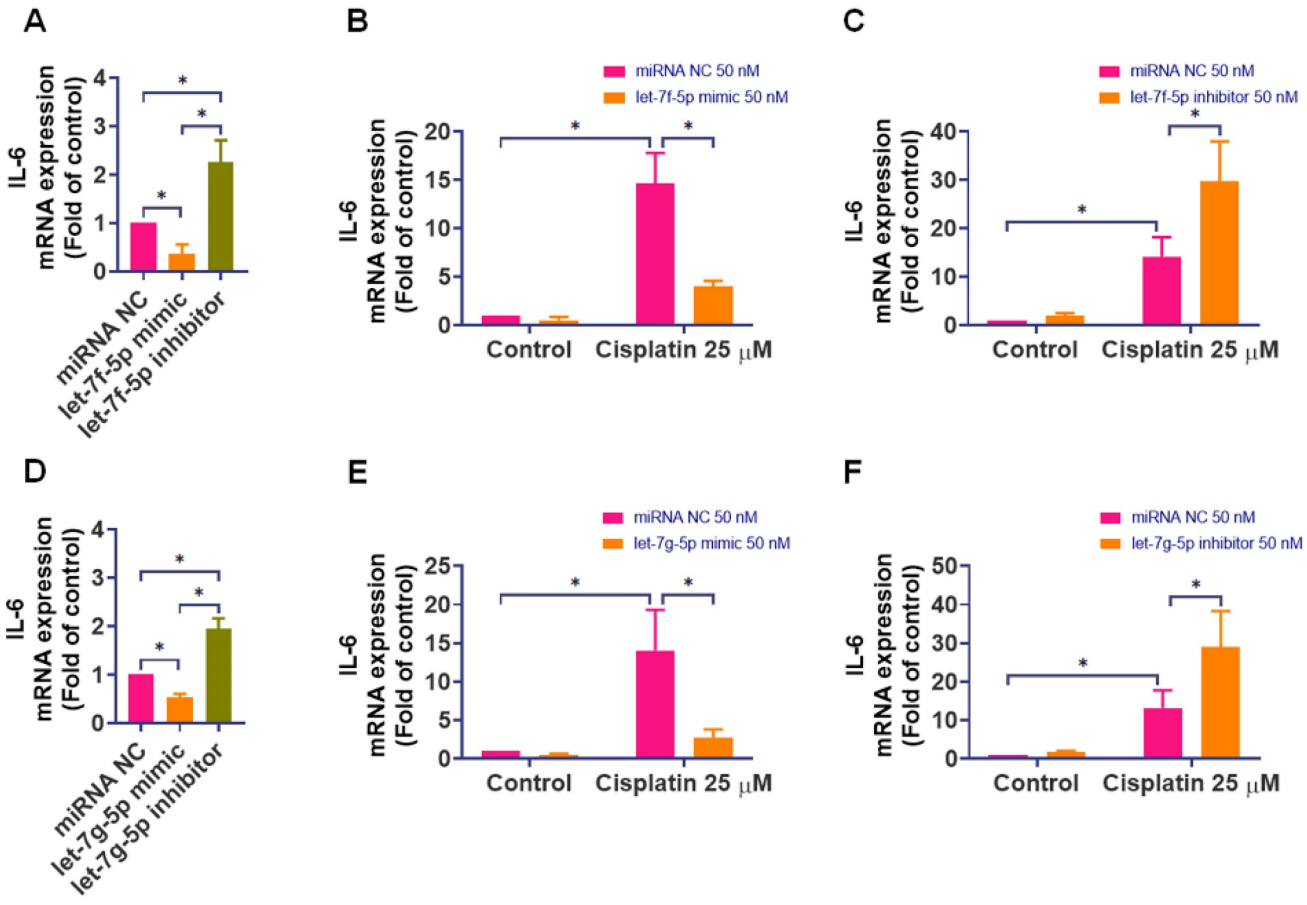
** The regulatory role of rno-let-7f-5p and rno-let-7g-5p on IL-6 expression in response to cisplatin.** IEC-6 cells were transfected with rno-let-7f-5p or rno-let-7g-5p mimics, inhibitors, or a negative control miRNA for 24-hour. **(A)** and** (D)** IL-6 mRNA expression was quantified using quantitative real-time PCR (n=4). **(B)** and **(E)** Following the transfection with miRNA mimics, cells were exposed to cisplatin (25 μM) for 24-hour. IL-6 mRNA levels were subsequently assessed (n=4).** (C)** and **(F)** After transfection with miRNA inhibitors, a similar cisplatin exposure was conducted, and IL-6 mRNA expression was measured (n=4). Untreated cells served as the control group. Results are expressed as means ± SD. Significance is denoted by *p < 0.05.

We utilized miRNA mimics and inhibitors to understand the specific targeting of rno-let-7f-5p and rno-let-7g-5p on IL-6. Transfecting IEC-6 cells with mimics of rno-let-7f-5p and rno-let-7g-5p (50 nM) significantly curtailed IL-6 expression, while their inhibitors markedly elevated it (Figure 4A and 4D). These results underscored the regulatory role of these miRNAs in IL-6 expression. Further, transfecting IEC-6 cells with mimics or inhibitors of rno-let-7f-5p and rno-let-7g-5p before 24-hour exposure to cisplatin revealed that the mimics significantly reduced cisplatin-induced IL-6 levels (Figure 4B and 4E), whereas the inhibitors significantly increased IL-6 expression induced by cisplatin (Figure 4C and 4F).

**Figure 5:**
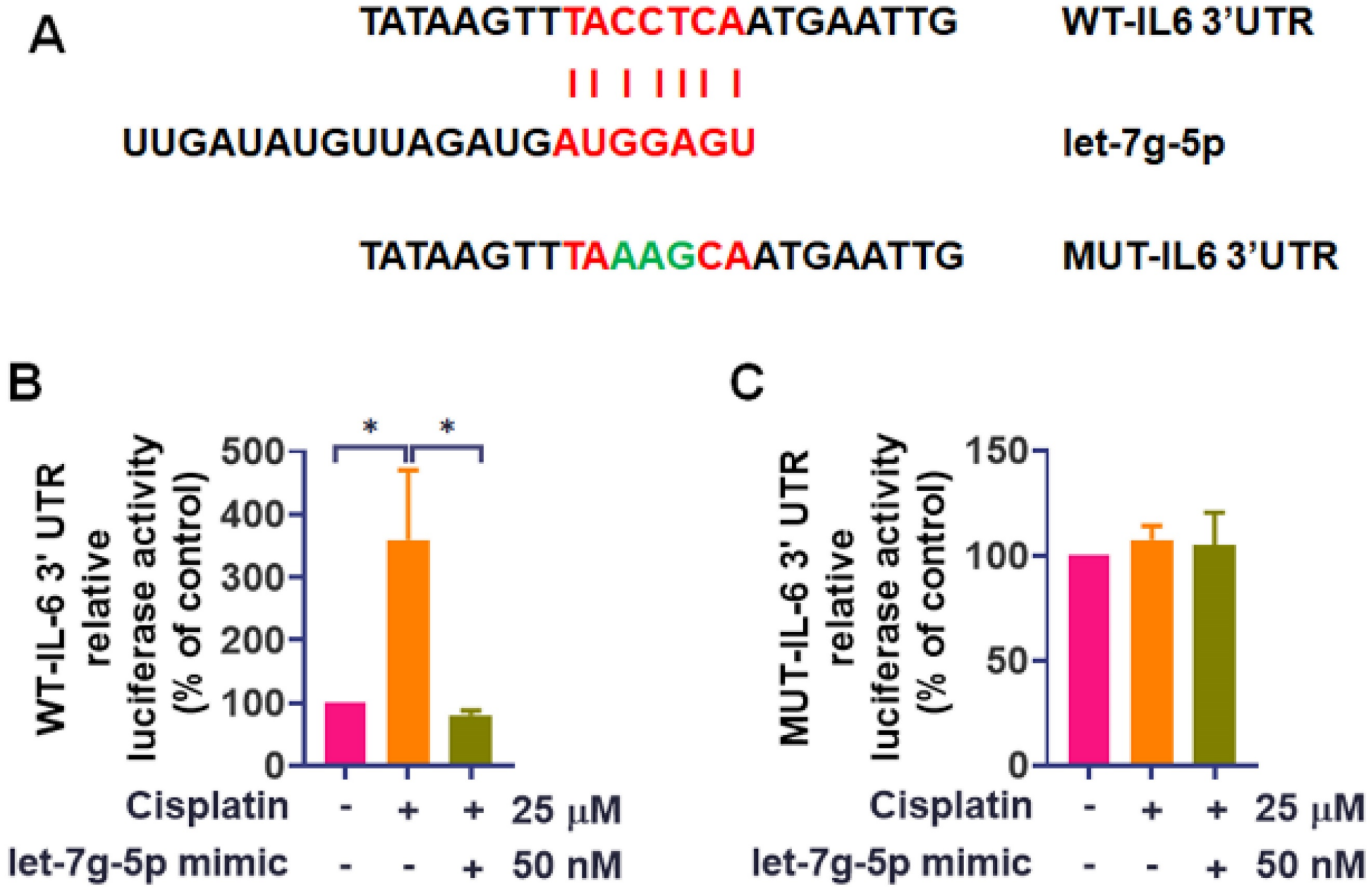
** rno-let-7g-5p directly binds to IL-6 3**'**UTR to reduce IL-6 synthesis. (A)** The schematic representation of the WT-IL-6 3'UTR and MUT-IL-6 3'UTR sequences is provided. IEC-6 cells were transfected with either IL-6 3'UTR wild-type plasmid or mutant IL-6 3'UTR plasmid, with or without the rno-let-7g-5p mimic for 24-hour. **(B)-(C)** Following transfection, cells were exposed to cisplatin (25 μM) for 24-hour. The relative luciferase activity, indicative of miRNA binding, was measured (n=4). Cells without any treatment served as the control group. Data are presented as means ± SD. Significance is denoted by *p < 0.05.

To further probe the mechanism, we employed luciferase reporter vectors containing either the wild-type 3'UTR of IL-6 mRNA (WT-IL-6-3'UTR) or a mutated vector with mismatches in the rno-let-7g-5p binding site (MUT-IL-6-3'UTR) (Figure 5A). Cisplatin treatment induced luciferase activity in cells with the WT-IL-6-3'UTR plasmid but did not affect the MUT-IL-6-3'UTR plasmid (Figure 5B-C). While the luciferase reporter assay directly confirmed binding of rno-let-7g-5p to the IL-6 3'UTR, rno-let-7f-5p was not examined in this assay in the current study. Nevertheless, its role in IL-6 regulation is supported by consistent gain and loss of function effects on IL-6 expression (Figure 4) together with bioinformatic prediction, and direct 3'UTR binding validation for rno-let-7f-5p will be addressed in future work. Remarkably, transfection of rno-let-7g-5p mimic diminished the transcriptional activity of WT-IL-6-3'UTR that cisplatin had increased (Figure 5B). These results collectively affirm the pivotal role of rno-let-7f-5p and rno-let-7g-5p in interfering with the IL-6 expression induced by cisplatin.

### Cisplatin decreases rno-let-7f-5p and rno-let-7g-5p levels in a p38- and ERK-dependent manner in intestinal epithelial cell

**Figure 6:**
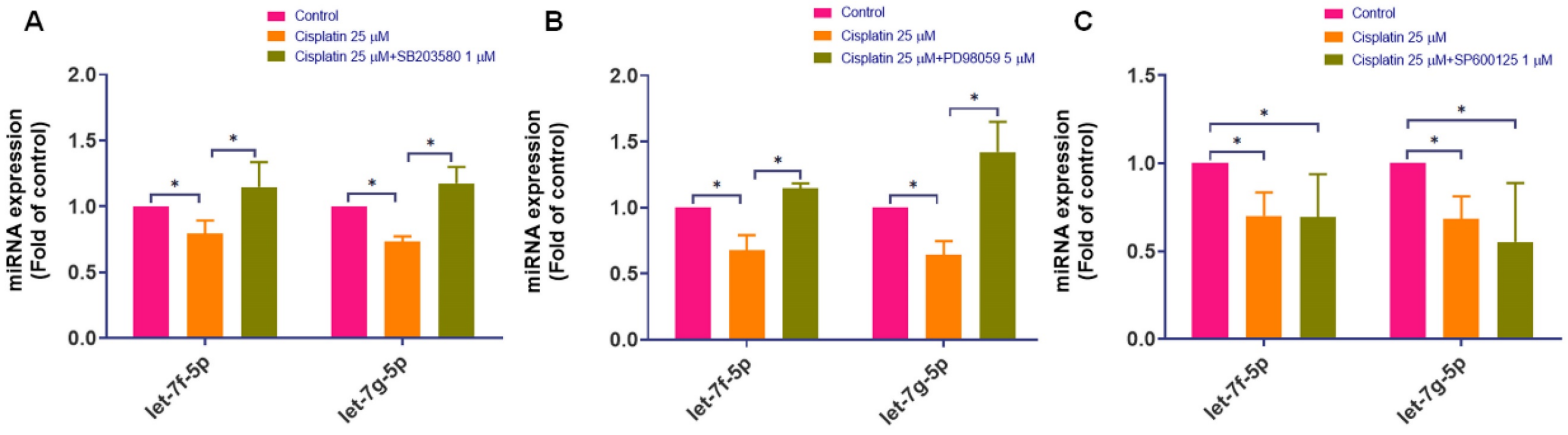
** The effect of cisplatin on the regulation of rno-let-7f-5p and rno-let-7g-5p through the MAPK pathway. (A)-(C)** Following the pre-treatment with p38, ERK, and JNK inhibitors for 1 h and exposed to cisplatin (25 μM) for 24-hour, the expression of rno-let-7f-5p and rno-let-7g-5p miRNAs was evaluated using quantitative real-time PCR (n=4). SB203580 (p38; 1 µM), PD98059 (ERK pathway; 5 µM), and SP600125 (JNK; 1 µM). Cells without any treatment served as the control group. Data are presented as means ± SD. Significance is denoted by *p < 0.05.

Given that cisplatin induces activation of the p38 and ERK pathways and increases IL-6 expression, we next assessed whether MAPK signaling is associated with the cisplatin-induced decrease in rno-let-7f-5p and rno-let-7g-5p. Inhibition of p38 or ERK significantly attenuated the cisplatin-induced reduction of rno-let-7f-5p and rno-let-7g-5p levels (Figure 6A-B). In contrast, JNK blockade did not reverse the cisplatin-associated decrease in these miRNAs (Figure 6C). Together, these results indicate that cisplatin-mediated downregulation of rno-let-7f-5p and rno-let-7g-5p is dependent on p38/ERK activity under our experimental conditions. Notably, we did not examine upstream transcription factors or pri-miRNA processing; therefore, these data support a signaling-dependent association rather than direct transcriptional regulation by MAPK pathways.

## Discussion

Our study showed that cisplatin treatment markedly increased IL-6 expression together with activation of the p38 and ERK pathways. By querying public miRNA target-prediction databases, we identified candidate miRNAs that may regulate IL-6 and found that cisplatin decreased the levels of rno-let-7f-5p and rno-let-7g-5p. Because upstream transcription factors, pri-let-7 transcripts, and miRNA biogenesis were not examined, our data do not establish direct transcriptional regulation of rno-let-7 by MAPK signaling. Nevertheless, integrating our signaling, inhibitor, and miRNA functional data supports a working model in which cisplatin activates p38 and ERK, suppresses rno-let-7f-5p and rno-let-7g-5p, and thereby de-represses IL-6 to promote an epithelial inflammatory response. This interpretation is supported by concordant pathway activation (p38/ERK phosphorylation), pharmacological inhibition (reduced IL-6 induction with partial restoration of rno-let-7 levels), and miRNA gain- and loss-of-function (mimic/inhibitor effects on IL-6), with direct IL-6 3'UTR binding validated for rno-let-7g-5p.

Importantly, our findings help bridge several lines of prior evidence that have largely been reported in isolation. Previous studies have shown that cisplatin can induce IL-6 and intestinal inflammatory responses, and that MAPK pathways contribute to inflammatory signaling 8-12, 18-22. Separately, members of the rno-let-7 family have been implicated in regulating cytokine expression, including IL-6, in other inflammatory settings 29, 30. However, the integration of these components into a single mechanistic framework within an intestinal epithelial cell model has remained limited. By combining MAPK pathway modulation with rno-let-7 functional perturbation and IL-6 readouts in IEC-6 cells, our study integrates cisplatin-triggered MAPK activation with rno-let-7-IL-6 regulation in a single epithelial model relevant to chemotherapy-associated mucosal injury. Thus, the key incremental contribution of this work is the integration of cisplatin-triggered MAPK activation and rno-let-7-IL-6 regulation into a single intestinal epithelial cell model relevant to chemotherapy-associated mucosal injury.

Cisplatin is unfortunately linked with numerous adverse effects that often necessitate dose reduction or discontinuation, thereby compromising its therapeutic efficacy 7. This challenge is not unique to cisplatin; other chemotherapeutic agents like 5-fluorouracil (5-FU) and irinotecan also elevate oxidative stress within the intestinal mucosa. Such oxidative stress triggers the release of inflammatory mediators, including IL-6, TNF-α, and IL-1β, which collectively contribute to the deterioration of the intestinal barrier and subsequent tissue damage 11, 31-33. Cisplatin has been frequently associated with intestinal inflammation and increased IL-6 expression 9, 10, 34. Moreover, the levels of IL-6 expression indicate disease progression and patient-specific characteristics in cases of intestinal ulcers 35. Our results corroborate these findings by demonstrating robust IL-6 upregulation in IEC-6 cells following cisplatin treatment, supporting IL-6 as a marker of epithelial inflammatory injury under these conditions.

miRNAs are pivotal in controlling various biological processes, primarily by hindering mRNA translation or hastening their degradation 36. Among these, specific miRNAs such as rno-let-7i-5p, rno-miR-98-5p, rno-let-7c-5p, and rno-miR-149-5p, including rno-let-7f-5p, have been identified as inhibitors of IL-6 expression, thereby curtailing the progression of inflammation 37-40. In this study, we used multiple prediction platforms to identify candidate miRNAs that may target IL-6, and prioritized nine overlapping candidates emerging from the intersection analysis. To delve deeper into the specificity of miRNAs in targeting IL-6 expression, we utilized miRNA inhibitors and mimics to substantiate the critical role of rno-let-7f-5p and rno-let-7g-5p in diminishing IL-6 expression within IEC-6 cells. The specificity of rno-let-7g-5p's interaction with the IL-6 3'UTR binding site was further validated through the transfection of IEC-6 cells with luciferase reporter vectors containing the wild-type or mutated-type 3'UTR of IL-6 mRNA. Consistently, cisplatin-induced activation of p38 and ERK was associated with decreased levels of rno-let-7f-5p and rno-let-7g-5p, and blocking p38/ERK partially restored these miRNAs. These findings prompt us to further explore the correlation between MAPK pathway activation and the expression of miRNAs in future studies.

The MAPK signaling pathway is a well-established mediator of inflammatory progression, as demonstrated in numerous studies 18-22. Activation of MAPK has been shown to suppress various anti-inflammatory miRNAs, facilitating the production of pro-inflammatory cytokines, including IL-6 41-43. The interaction between miRNAs and MAPK has garnered attention in different diseases. For instance, in Alzheimer's disease (AD), miRNAs such as miR-132 improve cognitive deficits in AD animal models by regulating the ERK/MAPK1 signaling pathway, affecting Aβ and Tau deposition and oxidative stress 44. Similarly, in ischemic stroke, miRNAs like miR-145, miR-137, miR-493, and miR-126 regulate apoptosis, neuroinflammation, neurogenesis, and angiogenesis through the MAPK pathway. Inhibition of MAPK activation attenuates the suppression of these miRNAs, suggesting potential neuroprotective therapies 45. Additionally, miR-29c targets and inhibits MAPK1, suppressing the activation of the ERK/MAPK pathway in pancreatic cancer. Restoring miR-29c expression inhibits the oncogenic phenotype of pancreatic cancer cells and reduces tumorigenic capacity *in vivo* 46. Consistent with these findings, our data demonstrates that cisplatin activates p38 and ERK, thereby enhancing IL-6 expression in intestinal epithelial cells. Pretreatment with p38 and ERK inhibitors reduced cisplatin-induced IL-6 mRNA expression and significantly mitigated the downregulation of the anti-inflammatory miRNAs rno-let-7f-5p and rno-let-7g-5p. Because pharmacological inhibitors may have off target effects, we interpret these results based on converging signaling and functional evidence: cisplatin increased p38/ERK phosphorylation, and p38/ERK inhibition reduced IL-6 induction while partially attenuating the decrease in rno-let-7f-5p/rno-let-7g-5p. Although additional pathways may contribute to IL-6 regulation, these data support a role for p38/ERK activity under our experimental conditions. We acknowledge that these data do not exclude potential JNK involvement at other doses, durations, or cellular contexts; however, no evidence of JNK activation or functional contribution was observed in our IEC-6 model under the conditions examined. These results suggest that cisplatin promotes inflammation, at least in part, through a MAPK activity-dependent decrease in specific rno-let-7 miRNAs, which may contribute to IL-6 upregulation.

To further clarify the translational relevance of our findings, it is important to discuss the cisplatin concentrations used in this study. Pharmacokinetic analyses indicate that peak plasma concentrations of unbound cisplatin in patients generally fall within the low micromolar range (approximately 1-10 μM), with C (max) values around 7.3 μM following clinical dosing 47. In contrast, higher concentrations such as 20-50 μM are commonly employed *in vitro* to reliably induce oxidative stress, MAPK activation, mitochondrial injury, and regulated cell death 48-51. Although these concentrations exceed typical circulating levels, they allow for reproducible modeling of acute epithelial injury. This discrepancy reflects a fundamental limitation of *in vitro* systems, which lack drug-binding proteins, multicellular interactions, and tissue-level pharmacokinetics. Notably, tissue exposure in the intestinal epithelium may differ from circulating unbound plasma concentrations due to local accumulation, repeated dosing, and epithelial susceptibility, but precise mucosal concentrations are difficult to infer from plasma measurements alone. Future studies using physiologically relevant doses, intestinal organoids, and *in vivo* models will help validate the p38/ERK-rno-let-7 regulatory axis under conditions that more closely mimic clinical exposure. In addition, the present study was performed in a monoculture IEC-6 system, which captures epithelial-intrinsic responses but does not recapitulate the multicellular and compartmentalized nature of intestinal inflammation *in vivo*. In the intestinal mucosa, epithelial cells communicate bidirectionally with immune cells (e.g., macrophages, neutrophils, and T cells), stromal cells, enteric neurons, and the microbiota, and these interactions can amplify or shape cytokine production and barrier disruption 52. Moreover, *in vivo* exposure involves complex pharmacokinetics and tissue distribution, as well as indirect inflammatory cues derived from damaged immune or stromal compartments. Therefore, our results should be interpreted as defining an epithelial cell-autonomous, MAPK-linked miRNA-cytokine axis that may contribute to mucosal inflammation, rather than fully modeling the *in vivo* inflammatory milieu. Future studies using co-culture systems, intestinal organoids, and animal models of chemotherapy-induced mucositis will be important to determine how the p38/ERK-rno-let-7-IL-6 axis operates in a more physiological context. To clearly delineate the boundaries of interpretation, we summarize key limitations below. Several limitations should be noted. First, as discussed above, this study was performed in a monoculture IEC-6 system, which captures epithelial-intrinsic responses but does not model immune-stromal-microbiota interactions that shape mucositis *in vivo*. Second, as discussed above, the cisplatin concentrations used enable reproducible epithelial injury *in vitro* but exceed typical unbound plasma levels, and mucosal exposure cannot be inferred from plasma measurements alone. Third, our conclusions regarding p38/ERK involvement rely on pharmacological inhibitors together with concordant signaling readouts; off-target effects cannot be fully excluded, and genetic approaches will further strengthen causality. Fourth, while rno-let-7g-5p binding to the IL-6 3'UTR was validated by luciferase reporter assays, direct binding confirmation for rno-let-7f-5p was not performed in this study. Finally, we did not assess upstream transcriptional regulators, pri-let-7 transcripts, or miRNA processing machinery (e.g., DROSHA/DICER), so the mechanism linking p38/ERK activity to rno-let-7 suppression remains to be defined.

Furthermore, although IL-6 was selected as the primary inflammatory mediator in this study, cisplatin is known to influence a broader spectrum of miRNAs and to induce diverse downstream functional consequences. Prior studies have identified miRNA-mediated regulation of apoptosis, oxidative stress adaptation, mitochondrial dysfunction, and drug resistance through pathways including miR-30c/SIRT1 and miR-101-3p/SOX2/ZIC5 53, 54. Cisplatin also triggers ferroptosis and pyroptosis in epithelial cells 48, 49, processes that may contribute to intestinal injury. In light of this, future investigations will include unbiased miRNA profiling, assessment of epithelial apoptosis, tight-junction integrity, and barrier-function assays such as TEER measurements, to provide a more comprehensive understanding of the epithelial responses to cisplatin.

To our knowledge, this study provides one of the first lines of evidence in an intestinal epithelial cell axis that cisplatin exposure is associated with a p38/ERK activity-dependent decrease in rno-let-7f-5p/rno-let-7g-5p levels alongside IL-6 upregulation, thereby linking MAPK signaling, rno-let-7 regulation, and IL-6 induction within a single experimental framework. These findings expand our understanding of the molecular mechanisms underlying cisplatin-induced gastrointestinal toxicity and highlight the p38/ERK-rno-let-7-IL-6 axis as a potential mechanistic target for future investigation. While these results establish an association between MAPK activation and decreased rno-let-7f-5p and rno-let-7g-5p expression, the transcriptional mediators that link p38/ERK signaling to miRNA repression remain unknown. Previous studies in related models have shown that activation of upstream kinases, including ERK and p38, can suppress specific miRNAs and thereby de-repress inflammatory or angiogenic effectors, supporting the plausibility of a kinase-to-miRNA regulatory axis 55-57. To establish causality, future studies will include: (1) ChIP-qPCR analysis of NF-κB/cAMP response element-binding protein (CREB) transcription factors at the rno-let-7f/rno-let-7g promoter regions; (2) quantification of pri-let-7 transcripts to determine transcriptional control; (3) actinomycin-D chase assays to assess transcription dependence; and (4) evaluation of DROSHA and DICER processing to distinguish transcriptional from post-transcriptional regulation 58, 59.

Although our study does not include direct therapeutic intervention experiments, accumulating evidence from other disease models supports the broader concept that modulating microRNA-signaling pathways can serve as an adjunct strategy. For example, thermomiR-377-3p enhances hyperthermia sensitivity in nasopharyngeal carcinoma by suppressing CIRBP 60; salvianolate mitigates doxorubicin/azithromycin cardiotoxicity through the miR-30a/BECN1 axis 61; and Huashi Jiedu Decoction increases 5-fluorouracil sensitivity by regulating the miR-21-3p/p53 pathway 62. Additionally, anti-miR-147 attenuates cold-storage transplantation injury via mitochondrial protection 63, and natural compounds such as baicalein, curcumin derivatives, and quercetin have been proposed as adjunct therapeutics in inflammatory and malignant diseases through miRNA and MAPK modulation 64-67. These studies support the conceptual relevance of targeting MAPK-regulated miRNAs as a complementary strategy, while underscoring that our current work provides mechanistic insight rather than direct therapeutic evidence. Future *in vivo* studies will be essential to determine whether modulation of the p38/ERK-rno-let-7f-5p/rno-let-7g-5p axis can mitigate cisplatin-induced intestinal inflammation and to evaluate the translational potential of miRNA- or MAPK-targeted interventions in gastrointestinal toxicity.

## Conclusion

This study identifies a cisplatin-responsive p38/ERK-rno-let-7f-5p/rno-let-7g-5p-IL-6 axis in intestinal epithelial cells. Under our experimental conditions, JNK signaling was not detectably activated, and JNK inhibition did not attenuate these responses. Inhibition of p38/ERK partially restores rno-let-7f-5p/rno-let-7g-5p and attenuates IL-6 induction, supporting this MAPK-linked miRNA axis as a mechanistically grounded framework for future strategies aimed at mitigating cisplatin-associated gastrointestinal toxicity.

## Supplementary Material

Supplementary figure.

## Data Availability

The datasets used and/or analyzed during the current study are available from the corresponding author on reasonable request.

## Abbreviations

IL-6: interleukin-6
IEC-6: intestinal epithelial cells
miRNA: microRNA
ERK: extracellular signal-regulated kinase
JNK: c-Jun NH2-terminal kinase
MAPK: mitogen-activated protein kinase
PCR: polymerase chain reaction
