# Supplementary material for: Cisplatin Induces Inflammation by Activating IL-6 via Suppressing rno-let-7g-5p and rno-let-7f-5p Expression in Intestinal Epithelial Cells
Source: Int J Med Sci. 2026 Mar 17;23(4):1554–66. doi: 10.7150/ijms.131043 (PMC13048891; doi:10.7150/ijms.131043)
Supplement: Supplementary file 1 — Supplementary figure. [file ijmsv23p1554s1.pdf]

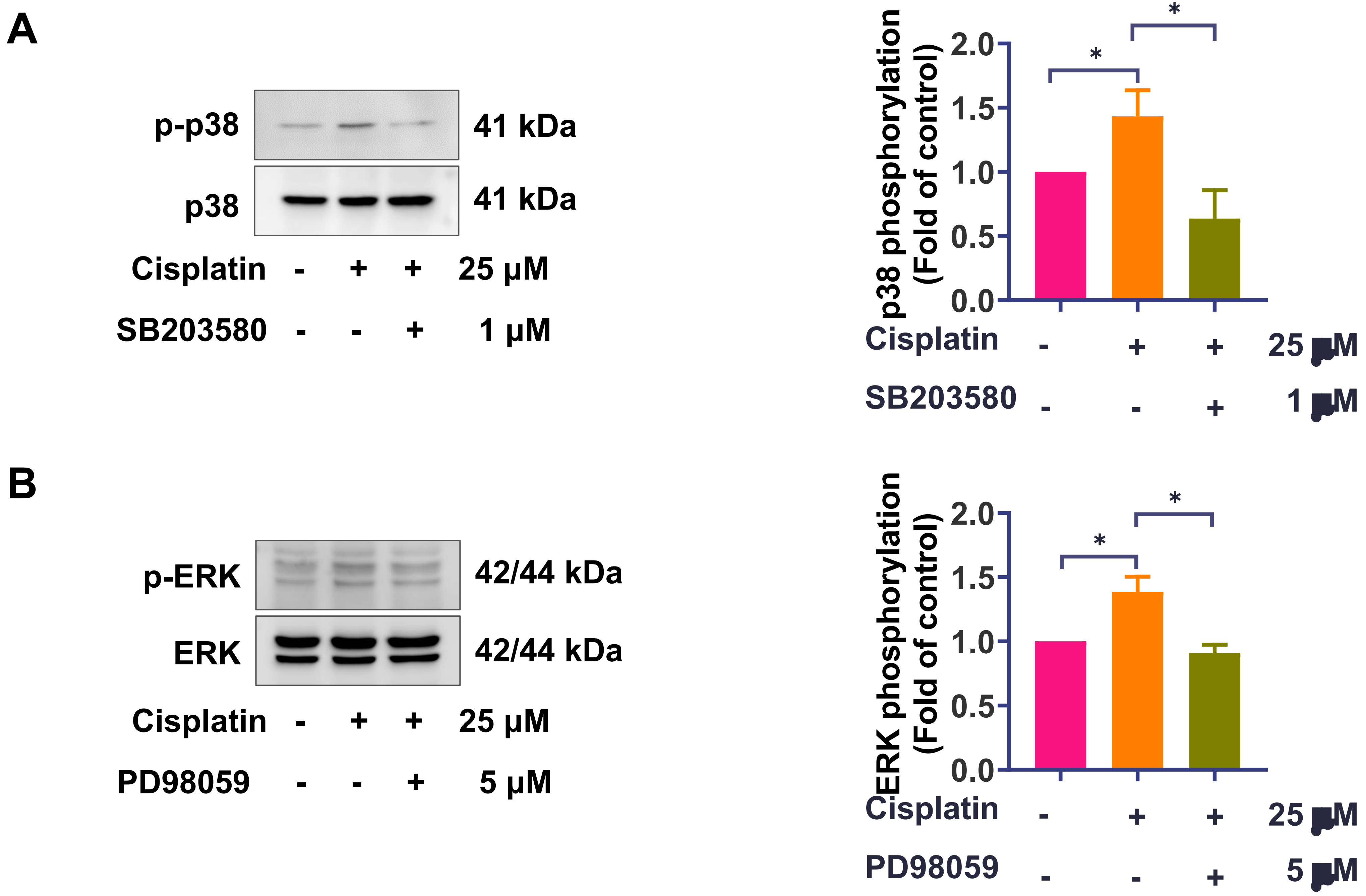
A
p-p38
41 kDa
p38
41 kDa
Cisplatin - + + 25 μM
SB203580 - - + 1 μM
p38 phosphorylation (Fold of control)
2.0
1.5
1.0
0.5
0.0
*
*
Cisplatin - + + 25 μM
SB203580 - - + 1 μM
B
p-ERK
42/44 kDa
ERK
42/44 kDa
Cisplatin - + + 25 μM
PD98059 - - + 5 μM
ERK phosphorylation (Fold of control)
2.0
1.5
1.0
0.5
0.0
*
*
Cisplatin - + + 25 μM
PD98059 - - + 5 μM

Supplementary Figure 1. Verification of p38 and ERK inhibitor efficacy. (A) IEC-6 cells were pretreated with the p38 inhibitor SB203580 (1 μM) for 1 hour and subsequently exposed to cisplatin (25 μM) for 30 minutes. Phosphorylated and total p38 levels were examined by Western blot analysis (n = 4). (B) IEC-6 cells were pretreated with the ERK pathway inhibitor PD98059 (5 μM) for 1 hour followed by cisplatin (25 μM) for 30 minutes. Phosphorylated and total ERK levels were assessed through Western blot analysis (n = 4). Untreated cells served as the control group. Data are shown as means ± SD. Statistical significance is indicated by $*p < 0.05$.
